# Evaluation of Endarterectomy Recanalization under Ultrasound Guidance in Symptomatic Patients with Carotid Artery Occlusion

**DOI:** 10.1371/journal.pone.0144381

**Published:** 2015-12-04

**Authors:** Yumei Liu, Lingyun Jia, Beibei Liu, Xiufeng Meng, Jie Yang, Jingzhi Li, Yinghua Zhou, Liqun Jiao, Yang Hua

**Affiliations:** 1 Department of Vascular Ultrasonography, Xuanwu Hospital, Capital Medical University, Beijing, China; 2 Department of Neurosurgery, Xuanwu Hospital, Capital Medical University, Beijing, China; Institute of Basic Medical Sciences, Chinese Academy of Medical Sciences, CHINA

## Abstract

Rigorous screening and good imaging would help perform surgery on carotid artery occlusion CAO safely and effectively. The purpose of this study was to retrospectively evaluate carotid endarterectomy (CEA) recanalization in patients with common carotid artery occlusion (CCAO) or internal carotid artery occlusion (ICAO) with color Doppler flow imaging (CDFI). A total of 59 patients undergoing CEA were enrolled. According to the results of CEA, the patients were divided into successful recanalization (group A) and unsuccessful recanalization (group B) groups. The original diameter, lesion length, proximal-to-distal diameter ratio and echo characteristics of the lesion within the lumen of the carotid artery were recorded before CEA and compared between the two groups. In regards to the achievement of repatency by CEA, the overall success rate was 74.6% (44/59), the success rate in CCAO patients was 75.9% (22/29) and the success rate in ICAO patients was 73.3% (22/30). There was no significant difference in the success rates between the CCAO and ICAO patients (χ^2^ = 0.050, *P* = 0.824). The overall rate of stroke and death within 30 postoperative days was 5.1% (3/59). For the CCAO patients, the lesion length in group A was shorter than that in group B (t = 3.221, *P* = 0.004). For the ICAO patients, the original diameter of the distal ICA was broader (t = 6.254, *P* = 0.000) and the proximal-to-distal ICA diameter ratio was smaller (t = 8.036, *P* = 0.000) in group A than in group B. The rate of recanalization for lumens with a homogeneous echo pattern (hypoecho or isoecho) was significantly higher than that for lumens with echo heterogeneity for both the CCAO and ICAO patients (χ^2^ = 14.477, *P* = 0.001; χ^2^ = 10.519, *P* = 0.003). However, for both the CCAO and ICAO patients, there was no difference in the rate of recanalization between patients with hypoecho and isoecho lesions (χ^2^ = 0.109, *P* = 0.742; χ^2^ = 0.836, *P* = 0.429). The original diameter, proximal-to-distal ICA diameter ratio, lesion length and echo characteristics may affect the success of CEA recanalization in patients with CCAO and ICAO. CDFI is helpful in screening patients with carotid artery occlusion and may improve the success rate of CEA.

## Introduction

Atherosclerotic carotid artery stenosis or occlusion is a leading cause of ischemic stroke. For patients with acute cerebral stroke caused by carotid artery occlusion (CAO), 40–69% of them develop permanent disability and 16–55% die, and only 2–12% patients have good prognoses [1]. For patients with CAO, 2%-5.5% experience a relapse of ipsilateral ischemic stroke annually [2].

The North American Symptomatic Carotid Endarterectomy Trial (NACET) of the treatment of patients with carotid artery near-occlusion showed that these patients benefit from CEA without an increased risk of stroke [3]. Some studies indicated that the risk of postoperative cerebral hemorrhage was increased by the performance of CEA in these patients [4, 5] due to the enhanced difficulty of the surgical technique, but other studies had different findings [6]. Using a simple method to assess the possibility and success rate of recanalization for the patients with carotid artery occlusion before CEA will be important for clinical decision making. As a non-invasive imaging technique, color Doppler flow imaging (CDFI) can be used to evaluate vessel structures and hemodynamics, which have been proven to aid preoperative screening, intra-operative evaluation and postoperative follow-up [7].

In this study, we aimed to retrospectively characterize the lesions within CAO and ICAO by CDFI, and further compare these characteristics between the group with successful recanalization and the group without recanalization.

## Materials and Methods

This study was performed after getting the approval of the Research Ethics Committee of the Xuanwu Hospital of Capital Medical University. All patients were fully informed of the procedure and signed a written consent form. The study was conducted in compliance with HIPAA regulations.

### Patients

From January 2008 to May 2015, a total of 981 patients with severe carotid artery stenosis or occlusion underwent CEA. All patients were evaluated by CDFI before undergoing CEA, and the CDFI findings were confirmed by digital subtraction angiography (DSA). A total of 59 patients with CCAO or ICAO were included in this study, and they underwent magnetic resonance imaging (MRI) or computed tomography (CT) before surgery to determine the presence of intracranial ischemic lesions.

The inclusion criteria were as follows: (1) patients with symptomatic CCAO or ICAO diagnosed by CDFI and confirmed by DSA and (2) ipsilateral ophthalmic artery image displayed on DSA. The exclusion criteria were as follows: (1) corresponding cerebral infarction 3 weeks prior to CEA; (2) any carotid occlusion associated with trauma, inflammation, or spontaneous dissection; (3) a severe bleeding tendency; and (4) serious surgical contraindications.

According to the results of CEA revascularization, patients were divided into two groups: successful recanalization (group A) and unsuccessful recanalization (group B) groups. The original diameter, lesion length, proximal-to-distal diameter ratio and echo characteristics within the lumen of the carotid artery were measured before CEA and were compared between the two groups.

### Ultrasound examination

**Fig 1:**
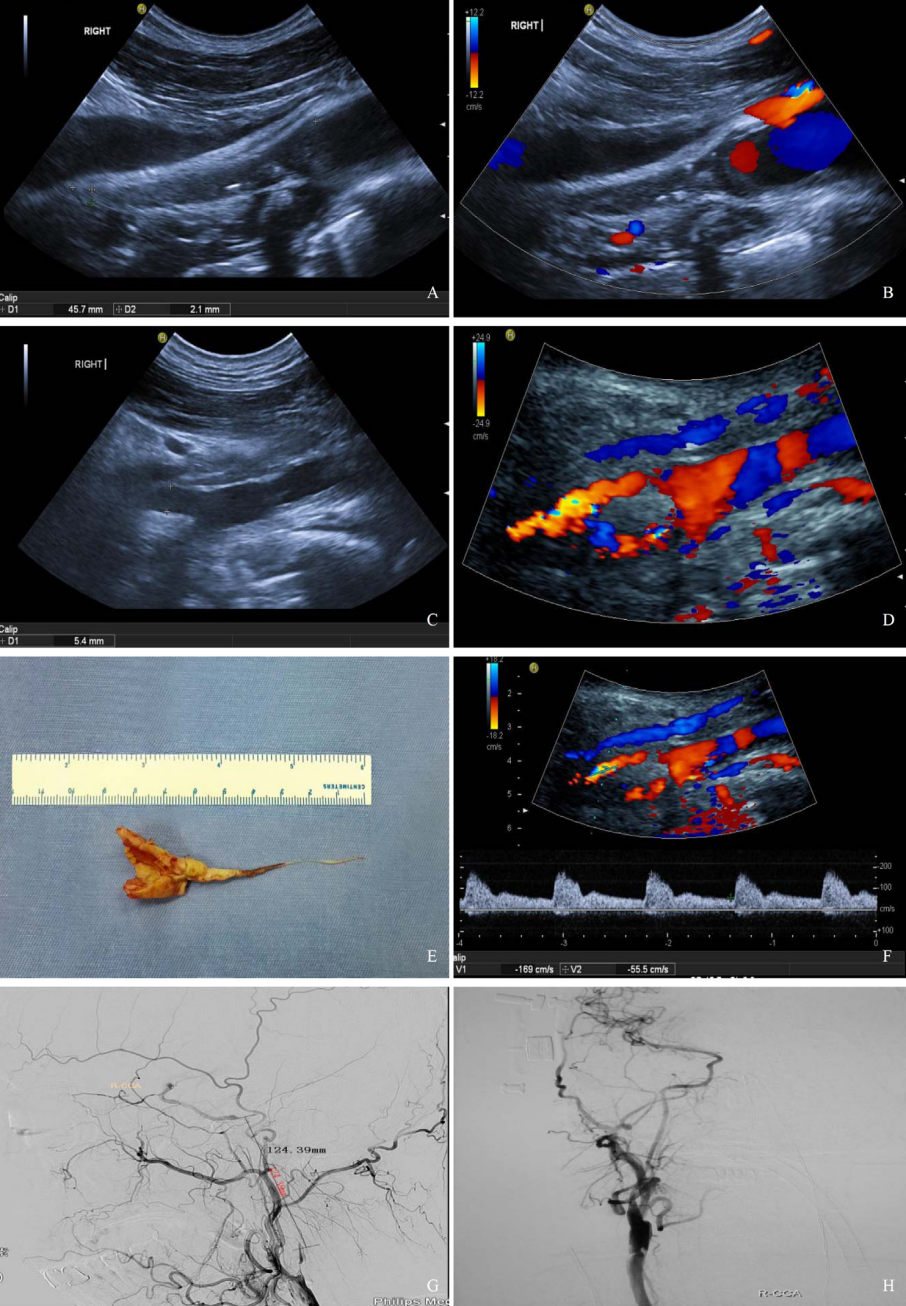
A 60-year-old male patient with right ICA occlusion underwent CEA. (A) Before CEA, two-dimensional ultrasonography image showing that the right ICA was filled with a hypoechoic lesion. For the original occlusion, the distal diameter was 2.1mm and the length of occlusion in the ultrasonic visual range was 45.7mm. (B) Color Doppler image showing no flow in the right ICA before CEA. (C) Two-dimensional ultrasonography image showing that the right ICA was reopened after CEA. The proximal diameter was 5.4mm. (D) Color Doppler image showing the blood flow filled in the right ICA after CEA. (E) The plaque and clot. (F) After CEA, the spectral wave form showing the peak systolic velocity of right ICA was 169cm/s, and the end diastolic velocity was 55.5cm/s. (G) Before CEA, DSA image showing occlusion of the right ICA and blood retrograde flow into the cavernous portion of the ICA. The occlusion length was 124.68mm. (H) After CEA, DSA image showing the right ICA was repatency.

CDFI was performed using iU22 (Philips) and Ascendus systems (HITACH) with broadband linear transducers (4.0–8.0MHz) and a convex array transducer (2.0–5.0 MHz) to evaluate the carotid artery before CEA. For presurgical evaluation by CDFI, the length (mm) of the lesion segment (the beginning to the end of the occlusion) of the CCAO or ICAO and the original diameters (mm) of the proximal and distal occlusion segment were measured, and the proximal-to-distal diameter ratio was calculated. The echo characteristics within the lumen detected by two-dimensional ultrasound were classified as hypoechoic, isoechoic, hyperechoic and heterogeneous echogenicity (Fig 1). In addition, the color Doppler ultrasonography was performed to confirm the recanalization within a week after CEA. When the blood flow filled in the carotid artery and the velocity increased to normal, the recanalization was deemed successful.

### DSA examination

Cerebral angiography was performed using the NEUROSTAR PLUS/T.O.P dual C-arm angiographic system (Siemens, Germany) both before and after CEA. The vessels above the aortic arch, such as the carotid, vertebral, subclavian and intracranial arteries, were examined. Images were taken in the lateral and double oblique projections. In all cases, detailed angiographic visualization of the entire anterior and posterior circulation was performed to confirm complete occlusion and to fully visualize the available collateral circulation. The length of the CCAO or ICAO segment was also measured (from the beginning to the end of the occlusion) before CEA (Fig 1).

### Surgical treatment

All CEAs were performed by experienced neurosurgeons under an intraoperative microscope (OPMI Pentero®, Germany). The revascularization procedure of CEA included plaque resection and arterial catheter embolectomy. When the blood flow of the ICA refluxed quickly after resection of the plaque and embolectomy, the recanalization was deemed successful and was confirmed by DSA. If no retrograde blood flow was observed, CEA recanalization was deemed to have failed.

### Statistical analysis

SPSS 22.0 software was used for the statistical analysis. The Chi-square test was used to analyze the differences in the risk factors of atherosclerosis and echogenic characteristics between the two groups. Student’s t-test was used to analyze the vascular diameter of the proximal and distal lesions and the proximal-to-distal diameter ratio in the subgroups. *P*<0.05 was considered statistically significant. (S1 Dataset)

## Results

### Patients’ characteristics

This study included 59 patients with a mean age of 61.0 (±8.5) years. No significant difference was observed in the risk factors for cerebrovascular diseases, such as hypertension, coronary heart disease, diabetes, and hyperlipidemia, between the two groups (Table 1). However, the CEA success rate was significantly lower in smokers. There was no significant difference between the two groups in the number of patients with clinical manifestations of TIA or stroke. Recanalization was achieved in 44 (74.6%) patients including one patient happened acute thrombosis within 6 hours who was performed repaired recanalization successfully by taking clot. Recanalization was not achieved in 15 (25.4%) patients. The rate of ipsilateral stroke or death within 30 postoperative days was 5.1% (3/59).

**Table 1:** Patients’ characteristics.

Factors	All patients n = 59 (%)	Group A n = 44(%)	Group B n = 15(%)	χ²	*P -*value
Age	61.0±8.5	61.3±8.3	60.0±9.0	—	0.619
Male	54(91.5)	40(90.9)	14(93.3)	0.085	0.771
Hypertension	31(52.5)	23(52.3)	8(53.3)	0.005	0.943
CHD	17(28.8)	12(27.3)	5(33.3)	0.200	0.654
Diabetes	22(37.3)	19(43.2)	3(20.0)	2.571	0.109
Hyperlipidemia	15(25.4)	13(29.5)	2(13.3)	1.551	0.213
Smoking	43(72.9)	28(63.6)	15(100)	7.484	0.006
TIA	29(49.2)	22(50)	7(46.7)	0.050	0.824
Stroke	30(50.8)	22(50)	8(53.5)	0.050	0.824

Note: Group A: successful recanalization; Group B: unsuccessful recanalization

### Presurgical ultrasonic characteristics of the occlusion segment

According to the lesion position, patients were classified into two subgroups: CCAO (29 patients) and ICAO (30 patients) subgroups, and the success rates for those subgroups were 75.9% (22/29) and 73.3% (22/30), respectively, and were not significantly different (*P* = 0.824).

For the CCAO patients, the original diameter of the lesion and the proximal-to-distal diameter ratio were not significantly different between group A and group B (Table 2). The lesion length in group A was shorter than that in group B demonstrated both by ultrasound and DSA (*P* = 0.004, *P* = 0.003). For lesions with a homogeneous echo pattern, the rate of recanalization was significantly higher than for those with a heterogeneous echo pattern (*P* = 0.001). There was no significant difference in the recanalization rate between the hypoecho and isoecho subgroups (*P* = 0.742).

**Table 2:** Preoperative sonographic features in the two subgroups of CCAO and ICAO patients.

	CCAO			ICAO		
Factors	Group A n = 22	Group B n = 7	*P-*value	Group A n = 22	Group B n = 8	*P-*value
Proximal diameter(mm)	6.5±1.1	6.2±1.5	0.468	7.5±1.4	7.7±1.4	0.727
Distal diameter(mm)	4.0±0.7	3.9±0.8	0.730	3.7±0.7	2.0±0.5	0.000*
Proximal/distal diameter ratio	1.6±0.3	1.6±0.2	0.538	2.1±0.6	4.0±0.6	0.000*
Occlusion length (CDFI, cm)	5.2±1.9	7.9±2.3	0.004*	4.2±0.4	4.2±0.5	0.981
Occlusion length(DSA, cm)	5.3±1.8	8.0±2.3	0.003*	7.1±1.6	6.9±1.1	0.707
Homogeneous echo	21(95.5%)	2(28.6%)	0.001*	19(86.4%)	2(25.0%)	0.003*
Heterogeneous echo	1(4.5%)	5 (71.4%)	0.001*	3(13.6%)	6(75.0%)	0.003*

Note: CCAO: common carotid artery occlusion; ICAO: internal carotid artery occlusion; Group A: successful recanalization; Group B: unsuccessful recanalization

*P<0.05

For the ICAO patients, the diameter of ICA in the original segment was not different between group A and group B (*P* = 0.727). However, the distal diameter of the ICA in the occlusion segment was apparently broader (*P* = 0.000) and the proximal-to-distal diameter ratio was smaller in group A (*P* = 0.000) compared to in group B. In no cases with an ICA distal diameter of less than 3 mm was recanalization successful. The length of the ICA occlusion examined by ultrasound and DSA was not different between the two groups (*P* = 0.981, *P* = 0.706). For lesions with a homogeneous echo pattern, the rate of recanalization was significantly higher than for those with a heterogeneous echo pattern (*P* = 0.003), and there was no significant difference in the rate of recanalization between patients with hypoecho and isoecho clots (*P* = 0.429).

## Discussion

Neuroimaging methods have played a crucial role in preoperative imaging examination and screening of CAO patients with acute ischemic stroke [8, 9, 10]. Computed tomography angiography (CTA), magnetic resonance artery (MRA), and DSA are widely used for the clinical diagnosis of CAO [8, 9, 10], but DSA, MRA and CTA only exhibit the loss of blood flow signals for the diagnosis of CAO [9, 10], without any information about the morphological characteristics of the blood vessel or the thrombus within the lesion lumen. CDFI has high diagnostic accuracy for CAO, reaching up to 96% [11]. In addition to identifying the treatment target, ultrasound vascular imaging can assist the neurosurgeon in formulating a treatment plan and in choosing appropriate tools based on multiple factors, such as level of occlusion, cervical steno-occlusive disease and vessel tortuosity. In this study, the lesion level and the length of the occlusion detected by ultrasonography were consistent with those detected by DSA.

Although it is controversial whether surgery should be performed in patients with symptomatic carotid artery occlusion, some studies have described the results of intra-arterial treatment for acute ischemic stroke in patients with extracranial ICA occlusions. In those studies, the overall recanalization rate was 60%-71% [8, 12], and the death rate was 11%-33% [8, 12, 13]. This study was retrospectively analyzed the reperfusion feasibility of symptomatic CCAO and ICAO. The success rate was 74.6% (44/59), and the incidence of stroke and death within postoperative 30 days was 5.1% (3/59).

Hugenholtz et al. [14] performed a study of thromboendarterectomy in 35 patients with symptoms distal and ipsilateral to an ICAO. They proposed that two major factors influence the choice of whether to perform surgery: the duration of occlusion prior to the potential time of surgery, when known, and the degree of collateral blood supply beyond the occlusion that flows retrograde into the distal ICA. In addition, Shucart et al.’s [15] report showed that there some chronic ICAOs exist that can technically be re-opened. If angiography shows retrograde flow into the carotid siphon and the patient’s symptomatology warrants surgical intervention, an attempt to reopen the ICA in the neck is warranted. Our study also showed that abundant collateral circulation maintained the patency of the carotid siphon, which may increase the success rate of thromboendarterectomy.

Recent studies have begun to focus on the impact of intravascular thrombus length on recanalization. Riedel et al. [16] reported that the length of occlusion can be used to predict patient outcome and the success rate of intravenous thrombolysis. This study also showed that patients with a shorter CCAO length had a higher rate of recanalization. When CCAO lesions are long, the difficulty of CEA recanalization and the risks associated with it are increased. For ICAO, this study showed lesion length was not significantly correlated with repatency rate, which is consistent with the view held by Hugenholtz et al. [14] and Shucart et al. [15]. It should be noted that although CDFI can only detect extracranial ICA, while DSA can detect the entire range of ICAOs, the two imaging techniques had similar predictive values for CEA repatency.

In addition to showing CAO length, the unique advantage of CDFI is that it can show the original diameter of the lesion. Archie JP Jr et al. [17] reported that patients with a normal diameter of the extracranial ICA had an excellent probability of successful CEA, but this was not the case when the distal artery was small or fibrotic. In our study, CDFI showed that blood flow after CCAO was reversed from the external carotid artery to the ICA, which resulted in the patency of the distal perfusion being maintained. At the same time, less atrophy occurred at the CCA lumen, and the original proximal and distal occlusion diameters were not significantly different. Furthermore, the proximal-to-distal diameter ratio was not correlated with the recanalization rate (Table 2). However, after ICA occlusion, with the extension of thrombosis to the distal end of the ICA, lumen diameter atrophy was often observed, and the thrombosis was more close to the vessel wall. Therefore, the distal diameter of the ICA detected by CDFI was usually small which may have attributed to the failure of recanalization. These findings were consistent with those of Archie JP Jr et al. [17]. Therefore, the diameter of the visualized distal extracranial ICA segment detected by CDFI may reflect the period of ICAO and predict the success of CEA.

Clot characteristics, including size and composition, may influence treatment success [18, 19]. Clot imaging has great value for surgery and may predict recanalization efficiency. Puig J et al. [20] used CT Hounsfield units to predict efficiency of recanalization of the intracranial artery according to clot composition. It is known that white thrombi consist of varying amounts of platelets, cellular debris and a few red cells, while red thrombi contain erythrocytes and some fibrin. Because clots with different histologic features can reflect the pathological process of carotid artery occlusion [21, 22], CDFI can also be used to estimate the duration of the clot’s presence with high accuracy based on echo characteristics [23]. In the acute stage, thrombi are always hypoechoic or isoechoic, while in the chronic stage, they are always hyperechoic [11]. In this study, we found that the rate of repatency was higher in patients with homogeneous echogenic (both hypoechoic and isoechoic) lesions than in patients with heterogeneous echogenic lesions visualized by CDFI. Hyperdense clots have been reported to be red blood cell-predominant, whereas low density clots have been reported to be fibrin-rich [24]. In animal models, red blood cell content has been shown to be positively correlated with the likelihood of successful recanalization with systemic thrombolysis [25]. Therefore, ultrasonic echo characteristics may reflect the duration of CCAO or ICAO and help predict the effectiveness of surgical revascularization.

## Conclusions

In summary, as a noninvasive, low-cost imaging technique, CDFI can provide detailed information that assists the diagnosis of carotid artery occlusion and surgical decisions for those patients. For patients with CCAO, the length of the occlusion may influence the recanalization rate. For patients with ICAO, a distal ICA with a small diameter and a large proximal-to-distal diameter ratio may risk the failure of procedure. In our cohort, the success rate was higher in patients with occlusions with homogeneous echo patterns than in patients with occlusions with heterogeneity echo patterns. The results need to be confirmed in another cohort that is done in prospective fashion. CDFI has a potential to be the imaging technique to screen patients with symptomatic CCAO or ICAO before being considered for CEA.

## Supporting Information

S1 Dataset The participant-level dataset. (SAV)
